# Bioactive compounds, antioxidant activity and physical characteristics of wheat-prickly pear and banana biscuits

**DOI:** 10.1016/j.heliyon.2019.e02479

**Published:** 2019-10-02

**Authors:** Lesetja M. Mahloko, Henry Silungwe, Mpho E. Mashau, Tsietsie E. Kgatla

**Affiliations:** Department of Food Science and Technology, School of Agriculture, University of Venda, Private Bag X5050, Thohoyandou, 0950, South Africa

**Keywords:** Food science, Food analysis, Flour, Biscuits, Composite flour, Total phenolic content, Antioxidant activity

## Abstract

In this study, banana and prickly peel flours were oven dried at 60 °C overnight and incorporated at a maximum of 4% (w/w) levels in wheat flour for biscuit production. Wheat, banana, prickly pear and composite flours and biscuits were evaluated for functional, bioactive compounds and antioxidant activities as well as physical properties. Functional properties analysis indicated that banana peel flour (BPF) and prickly pear flours (PPF) showed higher water holding capacity and oil holding capacity, ranging from 2.63 to 4.29 g/ml and from 1.15 to 2.0 g/ml, respectively. Total phenolic content ranged from 10.87 to 17.35 mg/g and from 11.21 to 11,44 mg/g in composite flour blends and total phenolic contents in biscuits improved from 11.365 mg/g to 11.81 mg/g with 4% BPF incorporation; and decrease to 10.92 mg/g with 4% PPF incorporation and 10.79 mg/g with 4% BPF and PPF, respectively. Total flavonoid content ranged from 15.78 to 23.19 mg/g in PPF and BPF, respectively and from 0.75 to 13.31 mg/g for control and composite flours. Moreover, results for Total flavonoid content of biscuits ranged from 17.0 to 33.74 mg/g. DPPH values ranged from 3.29 to 30.0% in flours and 8.12–9.69% in biscuits. FRAP values ranged from 0.57 to 1.51 mg/g for flours and 0.59–0.71 mg/g for biscuits. With regards to colour, incorporation of BPF and PPF resulted in decrease of L* value and b* values for composite flours and decreases in parameter L* and b* values for formulated biscuits. Spread ratio of biscuits showed an increase with addition of BPF and PPF, while diameter and height of biscuits decreased. Hardness of the biscuits increased with addition of BPF and PPF. Results suggest that by incorporating BPF and PPF, it is possible to enhance functional properties, colour parameters, antioxidant activity of the flours and biscuits.

## 1 Introduction

Biscuits are the most favoured and consumed bakery products globally due to the fact that they are ready to eat food, reasonable cost, high nutritional value, available in different flavour, taste and extended shelf life [1,2]. The production of acceptable quality biscuits relies on the selection of the correct flour and appropriate processing steps such as mixing, aeration, fermentation, baking, cooling and packaging [3]. Biscuits are considered as a type of confectionary with low moisture content and can serve as a vehicle tool for important nutrients if made readily available to the population [4,5]. Consumers’ awareness on the need to eat healthy and functional foods is increasing worldwide and health conscious consumers prefer food that furnish extra health benefits beyond the basic nutritional requirements [6,7]. Therefore, there is a trend to produce functional biscuits made from wheat flour and health promoting compounds from non-wheat flours known as functional ingredients [8]. The use of refined wheat flour and other ingredients result in biscuits lacking those components of grain that are intended to be preventative of health such as dietary fibre and phytochemicals [9,10]. Wheat flour on its own is a good source of calories and other nutrients but its antioxidant capacity is low due to refining during processing hence the need to composite it with prickly pear and banana peel flours to improve its antioxidant capacity. Prickly pear and banana peels are frequently regarded as by-products but are good sources of bioactive compounds and antioxidants [2,11].

Prickly pear (*Opuntia spp*) belongs to the family cactaceae and it is a wild fruit which grows in arid and semiarid regions and is widely distributed in Latin America, South Africa and the Mediterranean area [12]. The edible and non-edible parts of prickly pear plant contain phenolic compounds that have been reported to have many biological functions such as scavenging activity of harmful reactive oxygen species such as hydroxyl radical and hydrogen peroxide [13,14]. Bananas are mainly produced in tropical and subtropical countries. Banana flour is prepared by drying and grinding the ripe or unripe banana pulp. It is estimated that about 40% of total weight fresh banana fruit represents banana peel which is a good source of phenolic compounds. For instance, Fatemah *et al.* [11] in their studies found that banana peel contains phenolic compounds that range from 0.90 to 3.0 g/100g dry weight. Moreover, Kondo *et al.* [15] and Sulaiman *et al.* [16] showed that higher phenolic compounds are found in green banana peel as compared to the banana pulps and this has been extensively studied. Recent economical approach in maximizing exploitation of banana takes account of producing banana flour when the fruit is unripe and to integrate the flour into a variety of new products such as biscuits.

Recently, the food industry is dealing with high rate of food waste which is produced by fruit processing of different products such as juices, wines, jams, purees, etc [17]. Re use of prickly pear and banana processing waste, such as peel, could improve the yield of raw materials and subsequently minimise the large waste disposal problems faced by the food industry [18]. Therefore, the economic and technological feasible alternative will be to produce flours from both prickly pear and banana peels to make new products such as biscuits or to partially incorporate these flours in wheat flour in order to improve the nutritive value of biscuits since both fruits have good antioxidant potential and high in phenolic compounds and vitamin C.

Various studies have been carried out whereby wheat flour was replaced with flour from fruits waste or by-products to produce bakery products such as biscuits because of certain particular eating habits, new consumption trends, economic reasons and business requirements [19,20]. Elhassaneen *et al.* [2] reported the recovery and utilisation of prickly pear peel and potato peel by-products incorporated in crackers production improved bioactive compounds, dietary fibres and antioxidant activity. Therefore, products such as biscuits are a good subject to be studied on composite flours because of nutritional and economic reasons. However, it is necessary that the byproducts selected to be integrated in composite flours are assessed with regards to bioactive compounds and antioxidant activities for the development of technology that will be efficiently used in biscuits making without compromising the quality of products [17]. This study aims at developing and evaluating the wheat-prickly pear-banana peels composite biscuits, determining the bioactive compounds, antioxidant activity and physical properties of composite biscuits.

## 2 Materials and methods

### 2.1 Sourcing of raw materials

Wheat flour, margarine, bicarbonate soda, baking powder/yeast, salt, sugar, eggs and bananas were purchased from a local supermarket; prickly pears were sourced through a community project (Matoks, Limpopo province, South Africa). All chemicals (analytical grade) and reagents were purchased from Merck (Pty, Ltd South Africa).

### 2.2 Preparation of prickly pear flour

Ripe prickly pears were selected due to uniformity in size and colour, carefully and thoroughly washed then peeled with a knife to separate the peel pulp and seeds. The peels were dried at 60 °C for 24 h using hot air oven drier. Dried peels were ground into flour using a hammer mill.

### 2.3 Preparation of banana flour

Ripe banana fruits were washed and peeled, the pulps were discarded and the peels were cut into small pieces about 2 mm thickness. To prevent enzymatic browning, the banana peels were dipped in 0.5% (w/v) citric acid solution for 10 min, drained and dried in oven dryer (60 °C overnight). A hammer mill (Retsch ZM 81 200miller, Haan, Germany) was used to mill the dried peels at 16,000 rpm for 30 s to obtain banana peel flour. All banana peel flours were stored in airtight polyethylene bags at (10 ± 2 °C) until used [21].

### 2.4 Flour formulations

Composite flours were formulated according to the Table 1.

**Table 1:** Flour formulations.

Flour blend	Wheat flour (%)	Prickly pear flour	Ripe banana flour
WF	100%	-	-
BPWF	96%	4%	-
PPWF	96%	-	4%
BPPPWF	92%	4%	4%

WF = Wheat flour; BPWF = Banana peel and wheat flour; PPWF = Prickly pear and wheat flour; BPPPWF = Banana peel, prickly pear and wheat flour.

### 2.5 Preparation of biscuits

Biscuits formulations included flour (49.50%), margarine (20%), beaten whole egg (10%), sugar (20%) and baking powder (0.50%). The flour, sugar and baking powder were manually mixed into a bowl 500 cm^3^ because the quantity of the mixture was too small to use a laboratory mixer. Margarine and beaten whole egg were well creamed for 60 s then the dried ingredients were added at once and mixed for another 60 s. The batter was shaped using a round shaper (0.25 × 35 mm) and baked in an electric oven at 180 °C for 8 min. They were all allowed to cool on the table after which they were packaged in a low density polyethylene bag and kept in a plastic container for further analysis [4].

### 2.6 Determination of functional properties of formulated flours

#### 2.6.1 Water and oil-holding capacity

Water absorption capacity of flours was determined by the method of Anyasi *et al.* [22] whereby about 1 g of banana peel flour was weighed into 15 ml centrifuged tubes. Approximately 10 ml of distilled water was added to each sample and mixed thoroughly for 2 min and allowed to stand at room temperature for 30 min, then centrifuged for 20 min at 3000 centrifugal force. Water absorption was examined as per cent water bound per gram. The oil holding capacity (OHC) of the flours was also determined by the method of Anyasi *et al.* [22] whereby 1 g of banana peel flour was thoroughly mixed with 10 mL of cooking oil in 15 ml centrifuge tubes. The samples were allowed to stand for 30 min and centrifuged for 20 min at centrifugal force. Oil absorption was examined as percent oil bound per gram flour.

#### 2.6.2 Swelling power

The method of Tharise *et al.* [23] was used to determine the swelling power of flours whereby 50 ml centrifuge tube was used to weigh the transferred 0.1 g of sample. The distilled water was added to give a total volume of 10 ml. The sample in the tube was stirred gently by hand for 30 s at room temperature, and then heated at 60 °C for 30 min. After cooling to room temperature, the samples were centrifuged at 3000 rpm for 30 min. The weight of sediment was recorded.

#### 2.6.3 Bulk density

The method of Mariotti *et al.* [24] was followed to determine the bulk density of flours whereby 500 ml cylinder was filled with flour samples until the contents were tightly packed. A ratio between the sample weight and the volume of the cylinder was used to calculate the bulk density (g/ml3) of flours.

### 2.7 Total polyphenol content and antioxidant activity of flours and biscuits

Phenolic extracts were prepared by refluxing 2 g of each flour and biscuit sample with 20 ml of methanol containing 1% HCl for 2 h at 60 ± 5 °C [25]. The mixtures were centrifuged for 20 min at 5000 centrifugal force and the supernatants were separated and used for analysis of total phenolic and total flavonoid contents as well as antioxidant activity.

#### 2.7.1 Total phenolic content

The method of Singleton *et al.* [26] was used to determine the total phenolic contents of the sample extract whereby approximately 0.1 ml of the acidified methanolic extract was mixed with 5 ml distilled water in a 50 ml volumetric flask. Approximately 2.5 ml of Folin-Ciocalteu's reagent and 7.5 ml 15% sodium carbonate solution were added. The mixture was thoroughly mixed, made up to 50 ml and allowed to react for 30 min. A spectrophotometer (Biowave II, 80-3003-75, Biochrom LTD, Cambridge, UK) with a 96 well microplate was used to read the absorbance of the reaction mixture at 760 nm. A standard solution of gallic acid (R^2^ = 0.9993) was used to prepare a calibration curve and result was expressed as mg of gallic acid equivalent (GAE) per gram of the sample.

#### 2.7.2 Total flavonoid content

The method of Zhishen *et al.* [27] was adopted to determine the total flavonoid content of flours and biscuits whereby 0.1 ml of extract was mixed with 4.9 ml distilled water and 0.3 ml of NaNO_2_ was added. Approximately 0.3 ml of AlCl_3_ and 2 ml 1 M NaOH were added at 5 min and 6 min, respectively. The volume was made up to 10 ml with distilled water. The mixture was thoroughly mixed using the vortex equipment and the absorbance was read at 510 nm. A calibration curve was prepared using a standard of catechin hydrate (R^2^ = 0.9994) was used to prepare the calibration curve and the result was expressed as mg catechin equivalents per g of the sample.

### 2.8 Antioxidant activity

The method of De Ancos *et al.* [28] was used to assess the 1,1-diphenyl-2-picrylhydrazyl (DPPH) scavenging activity of flours and biscuits whereby about 10 μl aliquot of the acidified methanolic extract was mixed with 90 μl distilled water and 3.9 ml of methanolic 0.1 M DPPH solution. The mixture was thoroughly mixed by vortex equipment. The mixture was kept in the dark for 30 min and the absorbance was read at 515 nm. The result was expressed as percentage inhibition of the DPPH radical.

The method of Oyaizu [29] was followed to determine the ferric ion reducing antioxidant power (FRAP) the flours and biscuits whereby about 100 μl of the extract was placed in a test tube and methanol was used to adjust the volume to 1 ml. Approximately 2.5 ml 0.2 M phosphate buffer (pH 6.6) and 2.5 ml 1% potassium ferricyanide were added to the tube and thoroughly mixed with the vortex. The mixture was kept in water bath at 50 °C for 20 min. About 2.5 ml 10% (w/v) trichloroacetic acid was added to the mixture after incubation and centrifuged for 20 min at 5000 centrifugal force. About 2.5 ml of the supernatant was mixed with 2.5 ml distilled water and 0.5 ml 0.1% (w/v) ferric chloride in a test tube. The absorbance was measured at 700 nm. Higher absorbance indicates higher reducing power. Calculations were done using a standard curve prepared by ascorbic acid. Two antioxidants assay methods (DPPH and FRAP) were used because FRAP method is not able to detect slowly reactive polyphenolic compounds and thiols while the DPPH method is based on the ability of the stable 2,2-diphenyl-1-picrylhydrazyl free radical to react with hydrogen donors [30]. Therefore, one method cannot give scavenging of all free radicals. It would be more advantageous to determine the antioxidant capacity of a sample by more than one method.

### 2.9 Moisture and crude fibre contents

The moisture content of flours and biscuits was determined according to AOAC [31] method 945.32 with oven drying at 105 °C for 3 h. Crude fibre was determined by fibre-tech method according to AOAC [31] method number 985.33.

### 2.10 Determination of physical properties of the biscuits

#### 2.10.1 Colour analysis

The Minolta Spectrophotometer Model CM-3500d with a D65 light source (Minolta Ltd., Osaka, Japan) colour scale with the parameters L*a*b* was used to determine the colour of flours and biscuits. L* shows lightness, 0–100 with 0 indicating black and 100 indicating white. Coordinate a* corresponds to red (positive values) and green (negative values) while b* corresponds to yellow (positive values) and blue (negative values) [24], h° (hue) and C (Chroma) were recorded for each of the formulations.

#### 2.10.2 Diameter, height and spread ratio

Diameter (D) and height (H) was determined using Vernier caliper and it was used to calculate spread ratio with modifications [4]. Six biscuits were laid edge to edge and the overall diameter of the biscuits was measured. The biscuits were rearranged six times and the diameter recorded. Six biscuits were stacked at top of each other and the height of the biscuits was recorded using a Vernier caliper. The biscuits were rearranged six times and height recorded. The averages obtained were given off as diameter and height of the biscuits and values obtained used to calculate spread ratio as ratio of diameter of biscuits and height of biscuits.

#### 2.10.3 Texture analysis

The method of Chauhan *et al.* [32] was used to measure the hardness of baked biscuits using a texture analyzer (TA-XT2i, Stable Micro Systems, UK) in a compression mode with a sharp blade-cutting probe. The speed of pre-test, test and post-test were 1.5, 2, and 10 mm/s, respectively. For hardness, each sample was measured with more than six biscuits since hardness is a maximum peak force. The force–time plots were analysed for hardness or breaking force (g) to reach the peak.

### 2.11 Statistical analysis

Statistical analyses of all experimental data were analyzed using the statistical software package SPSS V.23 program. All comparisons were subjected to a one-way analysis of variance (ANOVA), and significant differences between treatment means were determined using Duncan's multiple range test at p < 0.05 as the level of the significance.

## 3 Results and discussion

### 3.1 Functional properties of wheat, banana and prickly pear peels flours

Functional properties of composite flours are shown in Table 2. Water holding capacity (WHC) ranged from 3.99 to 4.29 g/ml in banana and prickly pear peel flours and from 2.03 to 2.17 g/ml in wheat flour and composite flours. Addition of banana peel flour (BPF) and prickly pear flour (PPF) at 4% levels did not have any significant effect (p < 0.05) on WHC of composite flours. However, the WHC of BPF and PPF were significantly different (p < 0.05) as compared to composite flours.

**Table 2:** Functional properties of composite flours.

	WHC (g/ml)	OHC (g/ml)	SP (g/ml)	BD (g/ml)
BPF	4.29^c^ ± 0.17	2.01^c^ ± 0.37	7.06^b^ ± 1.52	0.62^a^ ± 0.04
PPF	3.99^b^ ± 0.15	1.95^c^ ± 0.37	7.80^d^ ± 0.49	0.61^a^ ± 0.01
WF (Control)	2.12^a^ ± 0.11	1.15^a^ ± 0.49	7.91^d^ ± 1.34	0.66^b^ ± 0.03
4%BPF96%WF	2.17^a^ ± 0.14	1.47^b^ ± 0.32	7.51^c^ ± 0.54	0.66^b^ ± 0.02
4%PPF96%WF	2.03^a^ ± 0.09	1.64^b^ ± 0.33	7.42^c^ ± 0.49	0.65^b^ ± 0.02
4%BPF4%PPF92%WF	2.14^a^ ± 0.07	1.90^c^ ± 0.10	6.19^a^ ± 0.32	0.66^b^ ± 0.04

Values are mean ± standard deviation of triplicate determinations. Values followed by the same superscript(s) within the same column are not significantly different at (p < 0.05). BPF = Banana peel flour; PPF = Prickly pear peel flour; WF = Wheat flour, WHC = Water Holding Capacity, OHC = Oil Holding Capacity, SP = Swelling Power, BD = Bulk Density.

Both BPF and PPF showed the highest WHC and this might be attributed to high presence of crude fibre (CF) available in both flours. Variations in the WHC of the flours could be due to difference in protein structure as well as the presence of different hydrophilic carbohydrates in BPF, PPF and wheat flour [33]. According to Alkarkhi *et al.* [23] dietary fibre, proteins and physical state of starch including the extent of fragmentation of native starch granules have an effect on WHC of flour. Similar trend in results was observed by Anwar and Sallam [34] where WHC of PPF was 1.80 g/ml and its addition into wheat flour numerically improved the WHC of the composite flour by range of 2–3% with only 2% partial replacement of wheat flour with PPF.

With regards to the oil holding capacity (OHC) of the flours, the composite flour from 4% BPF and 4% PPF and 92% wheat flour was significantly different from the control sample in OHC; however, both flours were not significantly different from 4% BPF and 4% PPF incorporation in wheat flour, respectively. OHC ranged from 1.95 to 2.0 g/ml in PPF and BPF, respectively and from 1.15 to 1.90 g/ml for control and composite flour blends. The control flour had the least OHC at 1.15 g/ml and flour composited with both PPF and BPF had the highest OHC. This could be attributed to variations in the presence of non-polar side chains which might bind the hydrocarbon side chains of oil in the flours [35]. Femenia *et al.* [36] observed that OHC depends on surface properties, overall charge density, thickness, and hydrophobic nature of the fibre particle, where those particles with the greatest surface area possess greater capacity of adsorbing and binding components of oily nature. Similar results were obtained by Anwar and Sallam [34] where partial replacement of wheat flour with 1 and 2% of PPF improved the OHC of flour ranging between 1 and 2.3 g/ml.

Swelling power ranged from 7.06 to 7.90 g/ml in BPF, PPF and wheat (control) flour and from 6.2 to 7.5 g/ml in composite flours. There was no significant difference (p > 0.05) in swelling power between control and composite flours. Addition of BPF and PPF did not improve the hydration capacity of the composite flour blends. The lower swelling power of composite flours might have been caused by higher amylose content and degree of intermolecular relationship in composite flours than in wheat, BPF and PPF flours. Bulk density ranged from 0.61 to 0.66 g/ml in PPF, BPF and wheat flour and from 0.65 to 0.66 g/ml in composite flours. There was no significant difference (p > 0.05) in BD among control and composite flours. Addition of BPF and PPF at 4% levels did not change the overall weight of composite flour blends. The highest BD of composite flour (4%BPF4%PPF92%WF) indicates that this flour can be used as thickener in food processing industry and it can also be used in food preparation because of its ability in helping to reduce the thickness of paste which is a predominant factor in convalescent and child feeding [37]. Increase in BD is desirable since it offers greater packaging advantage as a greater quantity of flour can be packed within a constant volume. Eltayeb *et al.* [38] reported similar findings on flour and protein isolate extracted from Bambara ground nut.

### 3.2 Moisture, bioactive compounds and antioxidant activity of flours and biscuits

Moisture, bioactive compounds and antioxidant activity of flours and biscuits are shown in Table 3. The moisture content of flour samples ranged from 8.02 to 3.64% with PPF having the least value. These low moisture contents of flours would be due to the efficiency of the drying methods used (Pierre, 1989). Indeed, it is well established that high moisture superior at 12% of food products promotes susceptibility to microbial growth and enzyme activity which accelerates spoilage (Brock et al., 1986; Ndangui, 2014; Anno et al., 2016) [39]. Moisture content is an index of storage of the flours. Flours moisture contents less than 14 % can resist microbial growth and contribute to best storage (Colas, 1998; Okonkwo and Opara. 2010). Similar observation was made by Matter (2015) [40] in wheat-sweet potato composite flours. This is advantageous because reduction in moisture content will reduce the proliferation of spoilage organisms especially mold, thus, improving the shelf stability of the product. The moisture content of biscuits ranged from 3.56 to 3.38% decreasing with increasing percentage of BPF and PPF substitution. The moisture content of the composite biscuits decreased with the addition of BPF and PPF, because the moisture content of the two flours was lower than that of wheat flour. Low moisture level is advantageous to the keeping quality of the biscuits since most spoilage microorganisms may not be able to survive at this moisture level (Agu and Okoli, 2014) [41]. The moisture content of the control and composite biscuits was within the recommended range of 0–10% for storage of biscuits (Singh et al. 2000) [42]. The shelf life of baked products has direct associations with their moisture content, which is an index of water activity and a measure of stability and susceptibility to microbial contamination. The present result is in agreement with the findings of Agu and Okoli [41] who found that the moisture content of biscuits decreased with the addition of beniseed and unripe plantain flour at different levels in the wheat flour. The result is also similar to findings by Bertagnolli et al. (2014) [17] who found moisture content ranging from 2.7 to 4.9% for wheat-guava peel biscuits.

**Table 3:** Moisture, crude fibre, total polyphenols and antioxidant activity of flours and biscuits.

	Moisture (%)	CF (mg/g)	TPC (mg/g)	TFC (mg/g)	DPPH (%)	FRAP (mg/g)
**Flours**						
BPF	5.57 ± 0.15	8.15^e^ ± 0.33	15.39^d^ ± 0.07	23.19^e^ ± 0.11	30.01^f^ ± 0.14	1.41^d^ ± 0.01
PPF	3.64 ± 0.08	6.37^d^ ± 0.13	17.35^e^ ± 0.02	15.78^d^ ± 0.03	11.15^e^ ± 0.07	1.51^d^ ± 0.01
WF	8.02 ± 0.01	1.17^a^ ± 0.17	10.87^a^ ± 0.07	11.69^b^ ± 0.05	4.89^b^ ± 0.07	0.57^a^ ± 0.01
4%BP96%WF	7.98 ± 0.05	2.49^c^ ± 0.18	11.20^b^ ± 0.04	0.75^a^ ± 0.03	3.29^a^ ± 0.08	0.71^c^ ± 0.01
4%PP96%WF	7.37 ± 0.18	2.36^c^ ± 0.13	11.38^c^ ± 0.02	13.22^c^ ± 0.03	10.39^d^ ± 0.04	0.65^b^ ± 0.01
4%BP4%PP92%WF	6.88 ± 0.12	2.02^b^ ± 0.02	11.44^c^ ± 0.09	13.31^c^ ± 0.03	9.25^c^ ± 0.09	0.70^c^ ± 0.00
**Biscuits**						
WFB	3.56 ± 0.08	0.69^a^ ± 0.07	11.37^b^ ± 0.05	18.39^b^ ± 0.05	9.61^c^ ± 0.11	0.59^a^ ± 0.01
4%BP96%WFB	3.49 ± 0.11	0.86^b^ ± 0.01	11.81^c^ ± 0.04	33.74^d^ ± 0.17	9.68^c^ ± 0.15	0.64^b^ ± 0.01
4%PP96%WFB	3.48 ± 0.10	1.36^c^ ± 0.09	10.92^a^ ± 0.03	19.01^c^ ± 0.04	9.16^b^ ± 0.07	0.63^b^ ± 0.00
4%BP4%P92%WFB	3.38 ± 0.22	2.13^d^ ± 0.07	10.79^a^ ± 0.08	17.04^a^ ± 0.11	8.12^a^ ± 0.11	0.70^c^ ± 0.00

Values are mean ± standard deviation of triplicate determinations. Values followed by the same superscript(s) within the same column are not significantly different at (p < 0.05). WF = Control Wheat flour, BPF = Banana peel flour; PPF = Prickly pear peel flour; 4%BP96%WF = Banana peel flour-Wheat flour composite; 4%PP96%WF = Prickly pear peel flour-Wheat flour composite; 4%BP4%PP92%WF = Banana peel-Prickly pear peel-Wheat flour composite; CF = Crude fibre; TPC = Total phenolic content; TFC = Total flavonoid content; DPPH = 2, 2-diphenyl-1-picrylhydrazyl, FRAP = Ferric ion reducing antioxidant power.

Crude fibre content ranged from 6.37 to 8.15 mg/g in BPF and PPF, respectively and from 1.17 to 2.49 mg/g in wheat flour and composite flours. BPF had significantly the highest crude fibre and PPF had lowest (p < 0.05) crude fibre. Similar trend was obtained by Alkarkhi *et al.* [23] and Emaga *et al.* [43] who indicated that banana peels are rich source of dietary fibre e.g. lignin (6–12%), cellulose (10–21%), hemicelluloses (6.5–9.4%) and galactouronic acid and their overall total dietary fibre content of banana peel was reported to range between 35 - 50%. The crude fibre content in PPF was higher than that found by El-Said *et al.* [44] and the differences could be due to the different growth conditions and stage of ripeness of the fruits. In this study, the crude fibre content was significantly different (p < 0.05) in all composite flours. Incorporation of BPF and PPF improved crude fibre content of the composite flours, however, incorporation of BPF and PPF at 4% levels in wheat flour was not significantly different (p > 0.05) from each other. Anwar and Sallam [34] reported that prickly pear peels on dry basis contain higher amounts of polysaccharides (25%), cellulose (29%) and hemi cellulose (8.5%). Moreover, Habibi *et al.* [45] also reported that prickly pear peels contain 2.4% lignin and 66% polysaccharides as well as 27% cellulose. The differences in cellulose and lignin contents of BPF and PPF could be the reason for the observed differences in crude fibre values since fibre tech method measures the cellulose and lignin contents of the flours. Similar results for crude fibre of PPF were reported by El-Said *et al.* [44] who observed 4.8 mg/100 g of crude fibre in prickly pear peels. The crude fibre of biscuits ranged from 0.69 to 2.13 mg/g in control wheat biscuits and composite biscuits. Control wheat biscuits had the lowest crude fibre content at 0.69 mg/g and combined incorporation of BPF and PPF at 4% levels into 92% wheat flour flour blend had significantly higher crude fibre content. Similar results for crude fibre of biscuits were obtained by Elhassaneen *et al.* [2] where incorporation of 5% prickly pear peel powder into wheat flour improved the total fibre content of the biscuits from 5.9 to 8.1 g/100 g, and from 5.9 to 8.7 g/100 g with 5% incorporation of potato peel powder.

Total phenolic content (TPC) of wheat-banana-prickly pear flours is presented in Table 3. TPC ranged from 15.39 to 17.35 mg/g in BPF and PPF and from 10.89 to 11.44 mg/g in wheat and composite flours. The control wheat flour had the lowest TPC and composite flour containing both BPF and PPF had the highest TPC. The composited flours were significantly different from the control wheat flour, however, there was no significant difference (p < 0.05) observed with addition of 4% PPF into 96% wheat flour and combined addition of BPF and PPF into 92% wheat flour. The high TPC values in PPF and BPF are attributed to 87.41% mg/100 g of ascorbic acid concentration in fruit peels per dry weight as stated by Anwar and Sallam [34] and Feugang *et al.* [46] who reported that fresh weight of prickly pears contain ascorbic acid content of 20–40 mg/100 g. Moreover, the fruit has other antioxidants which include pectin, carotenes, betalains, quercetin, and quercetin derivatives. According to Rebello *et al.* [47], banana fruits contain phenolic compounds such as catecholamines, phenolic acids and flavonoids. For both banana and prickly pear, the availability as well as the quantity of these health beneficial nutrients is influenced by various factors such as ripening stages of the fruit, location, climatic factor, agricultural and cultural practices [48,49].

The TPC of biscuits ranged from 10.79 to 11.80 mg/g with significant difference (p < 0.05) between control and composite biscuits. Addition of PPF and combined PPF and BPF significantly reduced (p < 0.05) TPC of biscuits and this could be due the sensitivity of vitamin C in both PPF and BPF to heat. Krystyjan *et al.* [50] indicated that the decrease of TPC is attributed to that baked products drastically reduce levels of phenolic compounds because of the epolymerization of polyphenols and decarboxylation of phenolic acids that occur during thermal treatment. Moreover, Gelinas and McKinnon [51] hypothesized the involvement of Maillard reactions to some degree in the content of phenolic compounds. The findings in the present study show a similar trend to studies by Elhassaneen *et al.* [2] where incorporation of prickly pear peel and potato peel powders at 5% level improved the TPC of the biscuits from 110.23 to 143.28 and 192.79 mg/100 g of sample. The results of total flavonoid content (TFC) of wheat-banana-prickly pear flours ranged from 15.78 to 23.19 mg/g in PPF and BPF respectively and from 0.75 to 13.32 mg/g for control wheat flour and composite flours. There was significant different at (p < 0.05) between BPF and PPF and they were also significantly different (p < 0.05) from control and composite flours. The high TFC in BPF as compared to PPF is owed to main classes of flavonoids detected in bananas which include quercetin, myricetin, kaempferol and cyaniding whereas prickly pear fruits are a rich source of flavonoids such as kaempferol, quercetin, narcissin, dihydrokaempferol [52]. Addition of PPF to wheat flour significantly improved TFC of composite flours whereas addition of PPF significantly lowered the TFC of composite flour blend.

Similar trend was obtained by Bamigbola *et al.* [53] where by addition of 27% plantain and 3% tigernut flour to wheat flour improved the TFC of wheat flour from 31.61 to 4.31 mg/g with wheat (70%), plantain (20%) and tigernut (10%) flour formulation and to 3.92 mg/g with wheat (65.66%), plantain (29%) and tigernut (5.33%) flour formulation however, the trend decreased to 3.25 mg/g with wheat (77%), plantain (20%) and tigernut (3%) flour formulation. The results for TFC of biscuits ranged from 17.04 to 33.74 mg/g. Biscuits formulated with combined incorporation of BPF and PPF had significantly low (p < 0.05) TFC at 17.04 mg/g and the significantly high TFC was observed in biscuits incorporated with 4% BPF with 33.74 mg/g. Baking contributed to a significant increase (p < 0.05) in TFC of the biscuits compared with the flours. The observed differences could be due to the development of meladoins which are brown pigments and are the products of Maillard reaction which occur during baking process [54]. Moreover, Pasqualone *et al.* [55] observed significant increase (p < 0.05) of flavonoids in biscuits and indicated that the increase was due to the contribution of semolina and shortening as well as incorporation of plant by products which imparts their volatile compounds.

DPPH of composite flours and biscuits is shown in Table 3. The DPPH ranged from 11.15 to 30.0% in PPF and BPF respectively and from 3.29 to 10.39% in control and composite flours. The DPPH was significantly different (p < 0.05) between control and composite flours. The DPPH of BPF was 30.0% which is within the range of those obtained by Fatemeh *et al.* [11] which ranged from 26.55 to 52.66%. Similar trend in this study was obtained by Bamigbola *et al.* [53] where addition of 27% plantain and 3% tigernut flour to wheat flour exhibited similar trend as observed in the study where the DPPH significantly decreased from 78.82 to 77.92%. Other parameters and the effect of the extracting solvent in dissolving endogenous compounds might have contributed to the behavior of obtained results of DPPH of flours [56]. The DPPH inhibition for plant materials normally follows a similar order of the TPC and TFC, for example, the DPPH increases when concentration of phenolic compounds or degree of hydroxylation of the phenolic compounds increases [57]. However, this was not the case in this study although BPF had the highest DPPH but its incorporation into wheat flour did not increase the DPPH of the flour blends. Similar trend was obtained by Fatemeh *et al.* [11] where the DPPH did not follow TPC and TFC order on BPF.

The DPPH ranged from 8.12 to 9.68% in control and composite biscuits. The lowest DPPH was observed in biscuits formulated with combined addition of BPF and PPF at 4% levels and the highest DPPH was obtained in biscuits formulated with 4% BPF. Baking resulted in 9.6 and 9.7% increase in 100% wheat flour and 4% of BPF. Sharma and Gujral [58] and Baba *et al.* [7] reported that processing steps such as baking and microwave roasting increase the antioxidant activity of baked products. This finding is consistent with a report by Jan *et al.* [35] where buckwheat flour was incorporated into wheat flour at 20 and 40% and resulted in improved % DPPH of composite flour from 55.53 to 57.18 and 61.65%, respectively.

FRAP values ranged from 1.41 to 1.51 mg/g in BPF and PPF, respectively and from 0.57 to 0.71 mg/g for control and composite flours. FRAP values showed significant difference (p < 0.05) in all composite flours and biscuits. The incorporation of BPF and PPF showed to improve the antioxidant activity of the flours. This indicates that the compounds present in the flour act more efficiently by the mechanism of hydrogen atom transfer than for electron transfer for ferric ion [50]. FRAP values of the biscuits ranged from 0.59 to 0.71 mg/g in control and composite biscuits. Composite biscuits still had significantly higher (p < 0.05) antioxidant activity than the control biscuits. These results are consistent with those reported by Jan *et al.* [35] where incorporation of buckwheat flour at 20 and 40% into wheat flour significantly improved the FRAP of composite flours compared to wheat flour, FRAP values improved from 33.15 to 37.30 and 41.07 mg/g in flours and from 27.16 to 45.5 and 49.82 mg/g in biscuits. Although lower TFC was observed in the flour when compared with the biscuits, however, higher antioxidant activities were found in the flour than the biscuits. The differences observed could be due to different structure of the phenolic compounds that might have influenced the antioxidant activities [59]. These phenolic compounds are easier to lose H atom that is able to scavenge the antioxidant assay. The antioxidant activity of the compound structure was reported to be dependable on the number of included active group (OH) and the position of the active groups [60].

### 3.3 Colour properties of wheat-banana-prickly pear flours

The colour of wheat-banana-prickly pear flours is shown in Table 4. The mean lightness (L*) value ranged from 29.47 to 34.63 in PPF and BPF, respectively and from 74.93 to 86.43 in formulated and control flours. There was a significant difference (p < 0.05) in terms of the lightness values of control and composite flours. The lowest L* value was in flour containing combined incorporation of BPF and PPF at 4% levels and the highest L* value was found in the control (wheat flour). Partial incorporation of BPF and PPF contributed to the significant decrease (p < 0.05) of the L* values of the wheat flour. This shows that there was a noticeable colour difference that existed between BPF and PPF flours and the composite flour formulations. Drying of the ripe banana peel resulted in major changes in colour of the flour yielding dark brown colour. The colour changes might have occurred due to the extent of Maillard reaction since banana peel contains glucose, fructose and protein [61]. Moreover, enzyme such as polyphenol oxidase may be present in banana peel that might contribute to enzymatic browning of the flours [62]. The latter explanation seems justifiable because enzymatic browning is a well-known problem in banana. The lightness (L*) values ranged from 41.57 to 65.0 in biscuits. The lightness values showed significance different (p < 0.05) between control and composite biscuits. The lowest L* value was observed in biscuits containing combined incorporation of BPF and PPF at 4% levels and the highest L* value was observed in control wheat biscuits. The observed differences in lightness could further be caused by heat during irregular exposure of the surface area of biscuits (in the oven) during baking, caramelization, and dextrinization process.

**Table 4:** Colour properties of wheat, banana and prickly pear flours and biscuits.

	L*	a*	b*	C	H°	ΔE
**Flours**						
BPF	34.63^b^ ± 0.473	6.03^d^ ± 0.252	13.23^f^ ± 0.306	14.53^d^ ± 0.404	65.533^d^ ± 0.289	52,27 ^d^ ± 0.19
PPF	29.46^a^ ± 0.513	23.33^e^ ± 0.058	0.07^a^ ± 0.058	23.37^e^ ± 0.057	0.200^a^ ± 0.100	62,46 ^e^ ± 0.26
WF	86.43 ^f^± 0.289	-0.20^a^ ± 0.100	10.17^e^ ± 0.153	10.17^c^ ± 0.153	91.233^f^ ± 0.551	-
4%BP96%WF	80.23^e^ ± 0.404	0.60^b^ ± 0.100	8.86^d^ ± 0.153	8.87^b^ ± 0.153	86.067^e^ ± 0.666	6,39 ^a^ ± 0.36
4%PP96%WF	78.50^d^ ± 0.529	3.63^c^ ± 0.252	5.03^b^ ± 0.153	6.17^a^ ± 0.306	54.300^b^ ± 1.054	10,20 ^b^ ± 0.29
4%BPPP92%WF	74.93^c^ ± 0.378	3.53^c^ ± 0.153	5.76^c^ ± 0.208	6.57^a^ ± 0.379	60.500^c^ ± 0.964	12,87 ^c^ ± 0.09
**Biscuits**						
WFB	65.000^d^ ± 0.529	4.067^a^ ± 0.058	27.533^d^ ± 0.757	27.833^c^ ± 0.757	81.633^d^ ± 0.416	
4%BP96%WFB	51.767^c^ ± 0.945	5.433^b^ ± 0.473	20.467^c^ ± 0.378	21.200^a^ ± 0.458	75.100^c^ ± 1.039	15,09 ^a^ ± 0.55
4%PP96%WFB	46.833^b^ ± 1.101	20.300^d^ ± 0.556	16.167^b^ ± 0.850	25.933^b^ ± 0.987	38.500^a^ ± 0.900	26,89 ^b^ ± 0.75
4%BPPP92%WFB	41.567^a^ ± 0.586	15.567^c^ ± 0.802	14.233^a^ ± 0.306	21.100^a^ ± 0.608	41.767^b^ ± 0.611	29,30 ^c^ ± 1.31

Values followed by the same superscript(s) within the same column are not significantly different at (p < 0.05). Values are mean ± standard deviation of triplicate determinations. BPF = Banana peel flour; PPF = Prickly pear peel flour; WF = Wheat flour; 4%BP96%WF = Banana peel-Wheat flour; 4%PP96%WF = Prickly pear peel-Wheat flour, 4%B4%P92%WF = Banana peel-Prickly pear peel-Wheat flour. L* = Lightness (+) blackness (-); a* = Redness (+) and greenness (*−*); b* = Yellowness (+) and blueness (*−*); Chroma* (colour intensity) and H° (hue angle colour saturation). **ΔE = T**otal colour change.

The a* values ranged from 6.03 to 23.33 in BPF and PPF, respectively and from -0.20 to 3.6 in control and composite flours. Control wheat flour showed the least a* values whereas wheat-prickly pear composite flour had the highest a* value. Moreover, the a* values ranged from 4.07 to 20.3 in the control and formulated biscuits. There was a significant difference (p < 0.05) with regards to a* values of control and composite biscuits. The significantly low a* values were observed in control biscuits at 4.07 whereas the significantly high a* values were observed in biscuits incorporated with 4% PPF. Yellowness (+) and blueness (−) (b*) values ranged from 0.07 to 13.23 in control flours and from 5.03 to 8.87 in composite flour blends. Wheat flour composited with 4% PPF had the least b* values and control wheat flour had the highest b* values. The high yellowness (b*) value in wheat flour is owed to yellow pigments xanthophylles often present in wheat flour. Partial substitution of wheat flour with both BPF and PPF significantly reduced (p < 0.05) the b* values of the composite flour blends. This could be attributed to the extent of browning in banana peel flour and high concentration of red pigments in PPF. Results of colour analysis show that at all levels of BPF and PPF addition, there was a significant decrease in parameter L* values and increases in parameter a* (greenness-redness) and b* (blueness-yellowness) values when incorporated with both BPF and PPF into wheat flour. The ripening of fresh banana during storage results in colour changes of the peel due to degradation of greenness with increase in reddish and yellowness tones [63] and this corresponds to increase in a* and b* values of BPF due to the degradation of the chlorophyll.

Yellowness (+) and blueness (−) (b*) values ranged from 14.23 to 27.53 in control and composite biscuits. The yellowness to greenness values for control and treatment biscuits were significantly different (p < 0.05). Biscuits composited with both BPF and PPF at 4% levels had the lowest b* values and control wheat biscuits had the highest b* values. The significant decrease in b* values could be attributed to the browning effect in BPF and betacyanins in PPF. The results are similar to the trend observed by Ho and Abdul-Latif [64] who observed a significant decrease (p < 0.05) in b* values of the composite biscuits ranging from 28.08 to17.38 with partial substitution of wheat flour with pitaya flour and amaranth respectively at 5, 10 and 15% levels of substitution. Furthermore, the PPF improved a* and b* values due to the high content of betalains. The results are similar to the trend observed by Ho and Abdul-Latif [67] who observed a significant decrease (p < 0.05) in b* values of the composite biscuits ranging from 28.08 to17.38 with partial substitution of wheat flour with pitaya flour and amaranth respectively at 5, 10 and 15% levels of substitution.

Chroma* (colour intensity) values of flours ranged from 14.53 to 23.37 in BPF and PPF, respectively and from 6.17 to 10.17 in control and composite flours and the chroma* of biscuits ranged from 21.10 to 27.83 in control and treatment biscuits. The chroma* of BPF and PPF showed significant difference at p < 0.05. Moreover, the chroma* results for control and composite flours were significantly different although flours and biscuits incorporated with 4% BPF and combined incorporation of BPF and PPF at 4% levels were not significantly different from each other but they were significantly different from control flour and biscuits. The chroma* values decreased in composite flours and biscuits with the incorporation of BPF and PPF; this observation can be explained by both chroma*, and H° being dependent on a* and b*.

The Hue angle H° (colour saturation) in flours ranged from 0.20 to 65.53 in PPF and BPF, respectively and from 54.30 to 91.23 in control wheat flour and composite flours; control and composite biscuits ranged from 38.50 to 81.63. Wheat flour and biscuits composited with 4% PPF showed the least values for H° and control had the highest H° values. The control flour and biscuits exhibited a pure yellow colour (90°), whereas the composited flour formulations with added BPF and PPF had an H° that tended toward pure red or pure magenta (0°). With regard to a* values, this parameter increased along with the increase in the percentage BPF and PPF in the composite flour formulations and biscuits. This was to be expected given that the PPF has a high content of betacyanins, which have colour with a greater red purple characteristic.

The colour difference values for BPF and PPF ranged from 52.27 to 62.46 and from 6.39 to 12.87 for composite flours while it ranged from 15.09 to 29.30 for biscuits. The colour difference of BPF and PPF as well as biscuits showed significant difference at p < 0.05. Biscuits fortified with 4% PPF had higher colour value (26.89) when compared with biscuits fortified with 4% BPF although the higher value was achieved in biscuits fortified with 4% BPF and PPF (29.30). The high colour difference value in biscuits could be due to the ingredient composition and red pigmentation resulting from the Maillard reaction or non-enzymatic browning which depends on the content of reducing sugars and amino acids or proteins on the surface, baking temperature, and time [65].

### 3.4 Physical properties of wheat-banana-prickly pear composite biscuits

The results for diameter of composite biscuits are shown in Table 5 ranging from 23.27 to 25.27 cm. The diameter of the biscuits showed a noticeable decrease with addition of BPF however; addition of PPF improved the diameter of the biscuits. The biscuits with 4% BPF and 4% PPF respectively were significantly different from control biscuits, however, biscuits composited with both BPF and PPF were not significantly different from control biscuits at p < 0.05. The observed shrinkage in diameter could be attributed to the crude fibre which tends to absorb moisture aiding development of gluten network forming an elastic network of the batter which after baking undergoes shrinkage following expansion [66]. Results observed in the study are consistent with the study by Asif *et al.* [64] who noticed a decrease in diameter of biscuits with addition of oven dried banana peel. Similar results were also obtained by Chinma and Gernah [4] where addition of 10% cassava flour and 10% soyabean flour improved the diameter of biscuits from 30.80 to 34.13 and 34.30 cm. The differences in values obtained could be due to the differences in percentages of plant flours incorporated in wheat flour.

**Table 5:** Physical properties of biscuits.

Biscuits	Diameter (cm)	Height (cm)	Spread ratio	Hardness (g)
WFB	24.30^b^ ± 0.20	9.01^b^ ± 0.03	2.69^a^ ± 0.02	2245.38^a^ ± 1.17
4%BP96%WFB	23.27^a^ ± 0.51	7.03^a^ ± 0.03	3.31^b^ ± 0.06	3572.83^d^ ± 1.14
4%PP96%WFB	25.27^d^ ± 0.40	6.93^a^ ± 0.12	3.65^c^ ± 0.08	3418.96^c^ ± 1.39
4%B4%P92%WFB	24.73^c^ ± 0.15	7.13^a^ ± 0.29	3.47^bc^ ± 0.16	2513.25^b^ ± 1.14

Values are mean ± standard deviation of triplicate determinations. Values followed by the same superscript(s) within the same column are not significantly different at (p < 0.05). WFB = Wheat flour biscuits; 4%BP96%WFB = Banana peel-Wheat flour biscuits; 4%PP96%WFB = Prickly pear peel-Wheat flour biscuits, 4%BP4%PP92%WFB = Banana peel-Prickly pear peel-Wheat flour biscuits.

The height of the biscuits ranged from 6.93 to 9.01 cm. Height of biscuits composited with BPF and PPF was significantly different (p < 0.05) from the height of the control biscuits. The control flour biscuits had ample availability of moisture imparted by baking ingredients resulting in a well-developed gluten network. The control biscuits had a significantly lower (p < 0.05) OHC as reduced fat binding capacity improves dough rise and height of biscuits. The lower height of biscuits could be attributed to the affinity of added BPF and PPF capacity to bind oil which retards proper gluten formation. Similar results were obtained by Chinma and Gernah [4] where addition of 10% cassava flour and 10% soyabean flour decreased (p < 0.05) the height of the biscuits from 40.0 in control biscuits to 36.10 and 36.21 cm, respectively. The overall spread ratio increased with addition of BPF and PPF. Spread ratio ranged from 2.69 to 3.65. Biscuits with 4% BPF and 4% PPF respectively were significantly different from control biscuits, however, biscuits containing both BPF and PPF did not show any significant difference from control biscuits (p < 0.05). The increase in spread ratio of the biscuits could be attributed to the OHC of the added plant by products which led to significant decrease in height of the biscuits [67]. Low spread ration in control biscuits indicates that starches in control (wheat flour) were more hydrophilic than the composite flours and this resulted in low spread ratio of the wheat (control) biscuits [68]. Moreover, increase in spread ratio of biscuits might also be an evidence of poor connection of protein and carbohydrates’ network in the biscuits and both protein and carbohydrate are important nutrients since they contribute to the hardness of the biscuits [69].

Low spread ration in control biscuits indicates that starches in control (wheat flour) were more hydrophilic than the composite flours and this resulted in low spread ratio of the wheat (control) biscuits [68]. Moreover, increase in spread ratio of biscuits might also be an evidence of poor connection of protein and carbohydrates’ network in the biscuits and both protein and carbohydrate are important nutrients since they contribute to the hardness of the biscuits [69].

Biscuits that have high spread ratio are regarded most desirable. This result is in line with the study by Nanyen *et al.* [67] who noted an increase in spread ratio with incorporation of wheat with Acha and Mung bean flour blends. Result obtained is also similar to study by Ho and Abdul-Lati [62] where addition of pitaya flour improved the spread factor of the biscuits.

The hardness of biscuits ranged from 2245.4 to 3572.8 g. The hardness of biscuits was significantly different across all treatments and control biscuits (p < 0.05). Biscuits with combined incorporation of 4% BPF and 4% PPF had significantly lower value of hardness compared to composite biscuits with 4% BPF and 4% PPF respectively. Control samples had the least hardness value. The differences observed might have been caused by the structure of the biscuits in addition to ingredients used which might generate large force fluctuations [70]. This could also be attributed to mixing which tends to distribute the added ingredients and this results in mixing supporting water absorption rather than developing a dough structure that is accurate. The expansion of gluten network is formed between glutenin and gliadin, contributing to the hardness of biscuits. Pareyt and Delcour [71] indicated that weight, hardness, density and stickiness of the dough can be reduced by high gluten levels. Results in this study are in disagreement with studies by Ho and Abdul-Latif [62] and Chauhan *et al.* [32] who observed a decrease in hardness of the biscuits with partial substitution of wheat flour with pitaya flour and amaranth respectively.

## 4 Conclusion

Flours and biscuits samples enriched with BPF and PPF by-products showed improved crude fibre, total phenolic compounds and flavonoids content than the control samples. Increasing of such bioactive compounds in BPF and PPF incorporated flours and biscuits, exhibited an improvement of their overall antioxidant activity and subsequently their properties as functional foods. Moreover, bulk density and swelling power of the flour incorporated with BPF and PPF remained unaffected whereas oil holding capacity and water holding capacity improved in composited flours. The physical properties of flours and biscuits portrayed variable results. The incorporation of BPF and PPF yielded variable pigmented composite flours and biscuits. The diameter and spread ratio of the biscuits increased with decrease in height.

## Declarations

### Author contribution statement

Mpho Mashau: Analyzed and interpreted the data; Wrote the paper.

Lesetja Mahloko: Conceived and designed the experiments; Performed the experiments.

Henry Silungwe: Conceived and designed the experiments; Analyzed and interpreted the data; Wrote the paper.

Tsietsie Kgatla: Analyzed and interpreted the data; Contributed reagents, materials, analysis tools or data; Wrote the paper.

### Funding statement

This work was supported by the University of Venda Research and Publication Committee, Thohoyandou, South Africa.

### Competing interest statement

The authors declare no conflict of interest.

### Additional information

No additional information is available for this paper.
